# The complete mitochondrial DNA genome of the Queensland seahorse *Hippocampus queenslandicus* Horne, 2001 (Gasterosteiformes: Syngnathidae)

**DOI:** 10.1080/23802359.2016.1225529

**Published:** 2016-12-09

**Authors:** Feixia Hou, Shasha Wang, Cheng Peng, Jinlin Guo

**Affiliations:** Pharmacy College, Chengdu University of Traditional Chinese Medicine, The Ministry of Education Key Laboratory of Standardization of Chinese Herbal Medicine, Key Laboratory of Systematic Research, Development and Utilization of Chinese Medicine Resources in Sichuan Province – Key Laboratory Breeding Base of Co-founded by Sichuan Province and MOST, Chengdu, China

**Keywords:** *Hippocampus queenslandicus*, mitochondrial genome, phylogenetic analysis

## Abstract

The complete mitochondrial genome of the Queensland seahorse *Hippocampus queenslandicus* Horne, 2001 (Gasterosteiformes: Syngnathidae) has been amplified and sequenced in this study. The mitogenome was 16,527 bp long with protein-coding genes, 2 ribosomal RNA genes, 22 transfer RNA genes and a non-coding control region. The overall base composition of the genomes was 32.2% for A, 30.1% for T, 14.9% for G, and 22.8% for C with an A + T-rich characteristic (62.3%). According to the phylogenetic analysis, *H. queenslandicus* showed a closer genetic relationship with *H. spinosissimus*.

The Queensland seahorse, *Hippocampus queenslandicus* Horne (2001) (Gasterosteiformes: Syngnathidae), is primary distributed in the north Queensland waters of the Great Barrier Reef, Australia (Horne 2001). *Hippocampus* queenslandicus is a deeper-water seahorse with a depth of 30–50m, usually located on a sponge or seagrass habitat, and attached to hard and soft coral species (Horne 2001). International trade of this species is monitored through a licensing system and a minimum size of 10 cm applies (Froese & Pauly 2016). However, the genetic information of *H. queenslandicus* has rarely been reported at present. In this study, the complete mitochondrial genome of *H. queenslandicus* was amplified and sequenced, which had been deposited in GenBank with accession no.KX685110.

The specimen was purchased from Chengdu Lotus Pond Herbal Medicine Market (Chengdu, Sichuan provice), and identified as *H. queenslandicus* based on its morphometric features and DNA barcoding technology. The sample used in this study was with Animal Ethics approval for experimentation granted by Chengdu University of Traditional Chinese Medicine. The complete mitogenome of H. *queenslandicus* was 16,527 bp in length, and contained 13 protein-coding genes, 2 rRNA genes, 22 tRNA genes, and a non-coding control region (D-loop), with identical gene arrangement and similar length as observed in other seahorse species (Chang et al. 2013; Song & Mabuchi 2014; Wang et al. 2014, 2015; Cheng et al. 2015; Zhang et al. 2015a, 2015b). Most of the genes were encoded on the heavy DNA strand (H-strand), excluding eight tRNA genes and ND6 gene. The overall base composition of the mitogenome was 32.2% for A, 30.1% for T, 14.9% for G, and 22.8% for C with an A + T-rich characteristic (62.3%) as that of other vertebrate mitochondrial genomes. All protein-coding genes, except for COI that was started with GTG, were initiated by ATG. Seven of 13 protein-coding genes were terminated with an incomplete stop codon T/TA, and six genes terminated with a complete TAA/TAG. The mitogenome of *H. queenslandicus* contained a 12S rRNA (937 bp) and a 16S rRNA (1698 bp), locating between tRNA-Phe and tRNA-Leu separated by tRNA-Val. The non-coding control region, which was 878 bp in length, was located between tRNA^Pro^ and tRNA^Phe^ genes. Among protein-coding genes, ND5 was the longest (1836bp), and ATP8 was the shortest (168 bp).

The phylogenetic analysis of 13 Syngnathidae species was performed using NJ algorithm based on the whole mitogenomes (Figure 1). The phylogenetic tree showed that *H. queenslandicus* had a closer genetic relationship with *H. spinosissimus* than other seahorse species.

**Figure 1.**
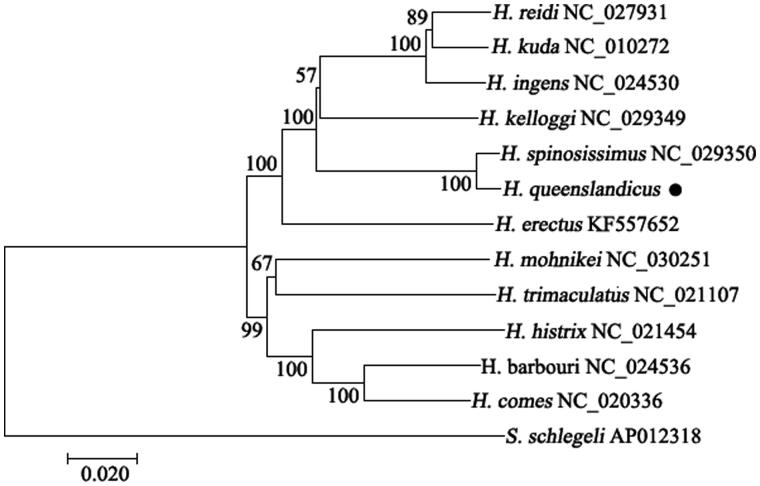
Phylogenetic tree of 13 Syngnathidae species constructed with the whole mitogenome using MEGA 7 (Temple University, Philadelphia, USA). *Syngnathus schlegeli* was used to the root the trees. Bootstrap values generated from 1000 replicates for NJ analysis.
